# Outcomes for Patients With Myeloid Neoplasms Treated With Chemotherapy Plus Venetoclax After Prior Venetoclax Therapy

**DOI:** 10.1002/jha2.70078

**Published:** 2025-06-13

**Authors:** Rafael Madero‐Marroquin, Joseph Cannova, Emily Dworkin, Austin Wesevich, Gregory W. Roloff, Caner Saygin, Mariam T. Nawas, Adam S. DuVall, Satyajit Kosuri, Michael J. Thirman, Olatoyosi Odenike, Wendy Stock, Richard A. Larson, Michael W. Drazer, Anand A. Patel

**Affiliations:** ^1^ Section of Hematology/Oncology Department of Medicine University of Chicago Chicago Illinois USA; ^2^ Department of Pharmacy University of Chicago Medicine Chicago Illinois USA

**Keywords:** acute leukemia, AML, chemotherapy, MDS, myeloid leukemia

## Abstract

**Background:**

Outcomes for patients with acute myeloid leukemia (AML) or myelodysplastic syndrome (MDS) that have progression after treatment with hypomethylating agent (HMA) and venetoclax (VEN) are poor. However, data for chemotherapy and VEN (C+VEN) therapy after prior treatment with HMA+VEN are limited.

**Methods:**

We identified 18 patients with AML or MDS/AML who received C+VEN after prior HMA+VEN.

**Results:**

Complete remission (CR) or CR with incomplete hematologic recovery (CRi) was achieved in 7 patients (39%) and 6 patients (33%) proceeded to allogeneic hematopoietic stem cell transplantation.

**Conclusion:**

This study shows suggests that C+VEN could be a viable option in a subset of patients after HMA+VEN.

## 1 Introduction

Outcomes for patients with relapsed/refractory (R/R) acute myeloid leukemia (AML) and higher‐risk myelodysplastic syndromes (MDS) are poor, particularly with disease progression after hypomethylating agent (HMA) and venetoclax (VEN) combination therapy. Additional effective treatment options are needed for this patient population. Intensive chemotherapy regimens have a reported overall response rate (ORR) of 28–62% in this setting, where there is no established standard of care [1, 2, 3]. While the addition of VEN to chemotherapy regimens has demonstrated high response rates both in the frontline and relapsed/refractory settings for patients with myeloid neoplasms [4, 5, 6, 7], data regarding the use of chemotherapy plus VEN (C+VEN) regimens after HMA+VEN failure are limited [8]. These data will become increasingly relevant as HMA+VEN is evaluated in the frontline setting in patients who are fit for intensive chemotherapy [9]. We present a retrospective study of patients with high‐risk myeloid neoplasms who were treated with C+VEN after previous treatment with HMA+VEN.

## 2 Methods

We identified patients ≥18 years old, with a diagnosis of a myeloid neoplasm, who were treated with C+VEN due to R/R disease after prior HMA+VEN therapy. This study was approved by our institutional review board prior to collecting data on patients’ clinical trajectories. Disease diagnoses were categorized according to the 2022 International Consensus Classification (ICC) of myeloid neoplasms [10]. Risk stratification and response assessment were performed according to the European Leukemia Net (ELN) 2022 criteria [11]. ORR was defined as the summation of cases with complete remission (CR), CR with partial hematologic recovery (CRh) and/or CR with incomplete hematologic recovery (CRi). OS and relapse‐free survival (RFS) were evaluated using the Kaplan–Meier method. ORR was also stratified by molecular characteristics, ELN 2022 risk stratification, line of therapy, *TP53*‐mutated status, history of prior allogeneic hematopoietic stem cell transplantation (allo‐HSCT), and by best response to initial treatment with HMA+VEN. OS was calculated from the time of C+VEN treatment initiation to the time of death. RFS was calculated for patients who had a remission; the duration was calculated from the time of remission to the time of relapse, death, or last follow‐up, whichever came first. Time to response was calculated from the time of C+VEN initiation to the time of best response to this therapy. Rates of major bleeding, febrile neutropenia, renal injury, hepatic injury, and tumor lysis syndrome (TLS) were calculated to assess C+VEN toxicity. Major bleeding was defined as intracranial hemorrhage or bleeding requiring management in an intensive care unit. Febrile neutropenia, renal injury and hepatic injury were defined using the 5^th^ version of the common terminology criteria for adverse events (CTCAE). Renal injury was defined using a cutoff of grade 3 or higher rise in serum creatinine. Hepatic injury was defined using a cutoff of grade 3 or higher rise in serum aspartate aminotransferase (AST), alanine aminotransferase (ALT), or bilirubin. Clinical and laboratory TLS rates were calculated using the Cairo‐Bishop criteria [12]. Given that most of the patients in our cohort (61%; *n* = 11/18) were treated with high‐dose cytarabine + mitoxantrone (HiDAC+MITO) + VEN as their C+VEN regimen, we compared ORR, median OS and rate of allo‐HSCT consolidation between HiDAC+MITO and HiDAC+MITO+VEN. Both cohorts were treated at our institution after previously receiving HMA+VEN. Prior lines of therapy, response rates and duration of response to prior lines of therapy were evaluated for each patient and this information was included in Table S1.

## 3 Results

**FIGURE 1:**
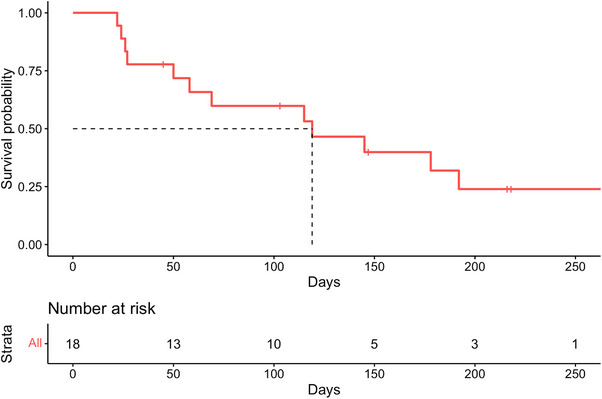
Overall survival from the time of treatment initiation with chemotherapy and venetoclax.

We identified a total of 18 patients, between February 2022 and November 2024, who met our inclusion criteria. The median age was 61 years [range 35–77]. Seventeen patients (94%; *n* = 17/18) had a diagnosis of AML and 1 (6%; *n* = 1/18) had MDS/AML prior to treatment with C+VEN. In combination with VEN, 61% (*n* = 11/18) of patients in our cohort received HiDAC+MITO, 22% (*n* = 4/19) received low‐dose cytarabine + cladribine (LDAC+CLAD), 6% (*n* = 1/18) received cladribine + high‐dose cytarabine + granulocyte‐colony stimulating factor (CLAG), 6% (*n* = 1/18) received CPX‐351, and 6% (*n* = 1/18) received fludarabine + high‐dose cytarabine + granulocyte‐colony stimulating factor + idarubicin (FLAG+IDA) [4, 5, 6]. Patients who received concurrent CYP3A4 inhibitors received a reduced VEN dose per the prescribing information. Patient and myeloid neoplasm characteristics for the C+VEN cohort are reported in Table 1. Most patients (72%; *n* = 13/18) had adverse risk disease by ELN 2022 criteria and were treated with C+VEN as third‐line therapy or later, while 50% (*n* = 9/18) had previously received intensive chemotherapy and 22% (*n* = 4/18) had a history of prior allo‐HSCT. Patients received a median of 2 cycles [range 1–11] of HMA+VEN prior to treatment with C+VEN with a median duration of 14 days [range; 7–21] of VEN during their first cycle of C+VEN. Patients with *KRAS*, *NRAS*, *FLT3*, or *TP53* mutations had limited responses to C+VEN (ORR 11%; *n* = 1/9). Three patients with a *KMT2A* rearrangement had received prior treatment with revumenib; 2 patients responded to C+VEN and proceeded to allo‐HSCT [13]. Our full cohort had an ORR of 39% (*n* = 7/18) with 33% of patients (*n* = 6/18) proceeding to allo‐HSCT after treatment with C+VEN. Median OS after starting C+VEN was 119 days (Figure 1). Patients who had an overall response had a median RFS of 136 days, and a median time to response of 39 days [range 33–53].

**TABLE 1:** Patient characteristics, myeloid neoplasm characteristics and treatment outcomes of patients treated with C+VEN.

	*N* = 18
Median age at the time of C+VEN therapy, years [range]	61 [35–77]
Sex, number (%)	
Male	13 (72%)
Female	5 (28%)
Race/ethnicity, number (%)^*^	
Non‐Hispanic white	13 (72%)
Non‐Hispanic black	1 (6%)
Hispanic	3 (17%)
ECOG PS at the time of C+VEN initiation, number (%)	
0	6 (33%)
1	10 (56%)
2	2 (11%)
Response to C+VEN therapy, number (%)	
CR	4 (22%)
CRi	3 (17%)
PR^#^	1(6%)
NR	10 (56%)
ORR to C+VEN by ELN 2022 risk classification, number of responses/total (%)	
Favorable	3/3 (100%)
Intermediate	0/2 (0%)
Adverse	4/13 (31%)
ORR to C+VEN by line of treatment, number of responses/total (%)	
2L	1/7 (14%)
≥3L	6/11 (55%)
ORR to C+VEN by molecular features, number of responses/total (%)	
*NRAS/KRAS*	1/5 (20%)
*TP53*	0/3 (0%)
*KMT2A*‐rearranged	2/3 (67%)
*NPM1* ^+^	2/2 (100%)
*FLT3‐*ITD	0/1 (0%)

* Information unavailable for 1 patient.

^#^ Had a CR after cytarabine‐based consolidation.

^+^ One patient's myeloid neoplasm also had an *IDH2* mutation.

*N* = number; C = chemotherapy; VEN = venetoclax; ECOG = Eastern Cooperative Oncology Group; PS = performance status; 2L = second line; 3L = third line; CR = complete remission; CRi = CR with incomplete hematologic recovery; PR = partial remission; NR = no response; ELN = European LeukemiaNet.

Fourteen patients (78%; *n* = 14/18) in this cohort were refractory to initial treatment with HMA+VEN and had an ORR of 43% (*n* = 6/14) with C+VEN. Median OS for this group was 117 days and HMA+VEN was the last line of therapy for 10 (*n* = 10/14) patients prior to C+VEN initiation. Three patients in this cohort (17%; *n* = 3/18) initially responded to HMA+VEN and had an ORR of 0% (*n* = 0/3) with C+VEN, although 1 patient had a PR and later achieved a CR with HiDAC consolidation. Median OS for this group was 58 days and HMA+VEN was the last line of therapy for all patients (*n* = 3/3) prior to C+VEN initiation. One patient received HMA+VEN as post‐transplant maintenance therapy and we did not categorize their response to HMA+VEN. Patients with a history of prior allo‐HSCT had an ORR of 75% (3/4) and a median OS of 192 days. Patients harboring a *TP53* mutation had an ORR of 0% (0/3) and a median OS of 119 days, while patients without a *TP53* mutation had an ORR of 47% (*n* = 7/15) and had a median OS of 115 days.

Most patients (89%; *n* = 16/18) experienced at least one adverse event during treatment with C+VEN regimens. Eighty‐nine percent (*n* = 16/18) of patients had febrile neutropenia, 28% (*n* = 5/18) had hepatic injury, 6% (*n* = 1/18) had renal injury and 6% (*n* = 1/18) had major bleeding. Eleven percent of patients (*n* = 2/18) experienced laboratory TLS and 6% (*n* = 1/18) experienced clinical TLS, which was treated with rasburicase. At the time of this analysis, 12 patients were deceased, of which 7 had R/R disease at the time of death; while 1 died due to chronic graft‐versus‐host disease (cGVHD), 1 from infection after allo‐HSCT, 1 due to infection during C+VEN induction, 1 from sagittal sinus thrombus, and 1 from post‐transplant lymphoproliferative disorder (PTLD). Four of the 6 surviving patients had allo‐HSCT after C+VEN.

We identified 15 patients at our institution who were treated with HiDAC+MITO after prior HMA+VEN. When compared to the 11 patients treated with HiDAC+MITO+VEN, the HiDAC+MITO cohort had a lower ORR (0%; *n* = 0/15 vs. 55%; *n* = 6/11), lower median OS (84 vs. 178 days) and a lower rate of allo‐HSCT consolidation (13%; *n* = 2/15 vs 45%; *n* = 5/11). One patient in the HiDAC+MITO cohort achieved a morphologic leukemia‐free state (MLFS). However, the HiDAC+MITO cohort had a higher proportion of patients with prior allo‐HSCT (40%; *n* = 6/15 vs. 27%; *n* = 3/11), more patients who received treatment as ≥ 3rd line of therapy (73%; *n* = 11/15 vs. 64%; *n* = 7/11) and a higher median age at the time of treatment (67 vs. 56 years) when compared to the HiDAC+MITO+VEN cohort.

## 4 Discussion

C+VEN yielded CR/CRi in 39% (*n* = 7/18) of patients in a heavily pre‐treated cohort previously treated with HMA+VEN. It was also a viable strategy towards allo‐HSCT consolidation for a subset of patients. Although 89% (*n* = 16/18) of patients experienced febrile neutropenia, as frequently seen with salvage chemotherapy, only 1 patient died of infectious complications during C+VEN induction. Only a small number out of the evaluable patients (18%; *n* = 3/17) had an initial response to HMA+VEN, consistent with a highly refractory cohort. Although the analysis was limited by sample size, the ORR was lower in patients who had an initial response to HMA+VEN when compared to patients who were refractory to prior HMA+VEN therapy. Higher response rates were observed in patients without *TP53, NRAS*, *KRAS* or *FLT3* mutations; which is consistent with reported outcomes using VEN in less intensive regimens, including HMA+VEN [14]. This regimen also yielded responses in patients harboring a *KMT2A*‐rearrangement with a history of revumenib therapy. Although C+VEN regimens have achieved high response rates in AML patients with a *KMT2A* rearrangement [15], to our knowledge, there are no data regarding the use of chemotherapy after HMA+VEN in this AML subset. Although further prospective data are needed to confirm the superiority of C+VEN over chemotherapy alone after HMA+VEN failure, the ORR seen with HiDAC+MITO+VEN suggests this regimen warrants further study as a tool to overcome VEN resistance. This treatment could prove to be particularly useful in the absence of targetable genetic aberrations.

## Conflicts of Interest

RM: no conflicts of interest to disclose. JC: no conflicts of interest to disclose. ED: honoraria from AbbVie. AW: no conflicts of interest to disclose. GWR: Advisory boards: Autolus Therapeutics and Kite/Gilead. CS: no conflicts of interest to disclose. MTN: no conflicts of interest to disclose. ASD: speaker for CE Concepts. SK: no conflict of interest to disclose. MJT: has acted as a consultant or advisor to AbbVie; AstraZeneca; Celgene; Janssen; Pharmacyclics; Roche/Genentech. Research funding from AbbVie (Inst); Gilead Sciences; Janssen, Merck; Nurix; Pharmacyclics; Syndax; TG Therapeutics. OO: institutional research funding by AbbVie, Astra Zeneca, Celgene, Curis, Incyte, Shattuck Lab, and K‐group alpha; scientific advisory board participant for AbbVie, Celgene/BMS, Novartis, Incyte, Kymera therapeutics, Servier, and Rigel; service on data safety board for Threadwell therapeutics. WS: advisor for Kura, Servier, Newave, and Asofarma. RAL: has acted as a consultant or advisor to AbbVie, Amgen, Ariad/Takeda, Astellas, Celgene/BMS, Curis, CVS/Caremark, Epizyme, Immunogen, Jazz Pharmaceuticals, Kling Biotherapeutics, MedPace, MorphoSys, Novartis, and Servier, and has received clinical research support to his institution from Astellas, Celgene, Cellectis, Daiichi Sankyo, Forty Seven/Gilead, Novartis, and Rafael Pharmaceuticals, and royalties from UpToDate. MWD: scientific advisory board, Argenx. AAP: honoraria from AbbVie, Bristol Myers Squibb, and Sobi; research funding (institutional) from Pfizer, Kronos Bio, and Sumitomo.

## Clinical Trial Registration

The authors have confirmed clinical trial registration is not needed for this submission.

## Supporting information




**Table S1**: Individual patient, disease and treatment characteristics of patients treated with C+VEN.

## Data Availability

Given concerns for medical privacy, data will not be made publicly available. For original data, please contact anand.patel@bsd.uchicago.edu.
