# Supplementary material for: Outcomes for Patients With Myeloid Neoplasms Treated With Chemotherapy Plus Venetoclax After Prior Venetoclax Therapy
Source: EJHaem. 2025 Jun 13;6(3):e70078. doi: 10.1002/jha2.70078 (PMC12163342; doi:10.1002/jha2.70078)
Supplement: Supplementary file 1 — Table S1: Individual patient, disease and treatment characteristics of patients treated with C+VEN. [file JHA2-6-e70078-s001.docx]

**Table S1. Individual patient, disease and treatment characteristics of patients treated with C+VEN**

**Table S1 legend:**

C= chemotherapy; VEN= venetoclax; CR= complete remission; CRi= CR with incomplete hematologic recovery; PR= partial remission; NR= no response; ELN= European LeukemiaNet; allogeneic hematopoietic stem cell transplantation=allo-HSCT, DEC= decitabine; AZA= azacitidine; DEC-CE= decitabine-cedazuridine; OS= overall survival; GO= gemtuzumab ozogamicin; NK= natural killer; HiDAC+MITO= high-dose cytarabine + mitoxantrone; LDAC+CLAD= low-dose cytarabine + cladribine; CLAG= cladribine + high-dose cytarabine + granulocyte-colony stimulating factor; CLAG-M= CLAG + mitoxantrone; FLAG+IDA= fludarabine + high-dose cytarabine + granulocyte-colony stimulating factor + idarubicin; 7+3= cytarabine + daunorubicin.
